# LncRNA Pathway Involved in Premature Preterm Rupture of Membrane (PPROM): An Epigenomic Approach to Study the Pathogenesis of Reproductive Disorders

**DOI:** 10.1371/journal.pone.0079897

**Published:** 2013-11-27

**Authors:** Xiucui Luo, Qingxi Shi, Yang Gu, Jing Pan, Maofang Hua, Meilin Liu, Ziqing Dong, Meijiao Zhang, Leilei Wang, Ying Gu, Julia Zhong, Xinliang Zhao, Edmund C. Jenkins, W. Ted Brown, Nanbert Zhong

**Affiliations:** 1 Center of Translational Medicine for Maternal and Children's Health, Lianyungang Maternal and Children's Hospital, Lianyungang, Jiangsu, China; 2 Hunter College High School, New York, New York, United States of America; 3 New York State Institute for Basic Research in Developmental Disabilities, Staten Island, New York, United States of America; 4 Peking University Center of Medical Genetics, Beijing, China; 5 Shanghai Children's Hospital Affiliated to Shanghai Jiaotong University, Shanghai, China; 6 March of Dimes Global Network of Maternal and Infant Health, White Plains, New York, United States of America; VU University Medical Center, The Netherlands

## Abstract

Preterm birth (PTB) is a live birth delivered before 37 weeks of gestation (GW). About one-third of PTBs result from the preterm premature rupture of membranes (PPROM). Up to the present, the pathogenic mechanisms underlying PPROM are not clearly understood. Here, we investigated the differential expression of long chain non-coding RNAs (lncRNAs) in placentas of PTBs with PPROM, and their possible involvement in the pathogenic pathways leading to PPROM. A total number of 1954, 776, and 1050 lncRNAs were identified with a microarray from placentas of PPROM (group A), which were compared to full-term birth (FTB) (group B), PTB (group C), and premature rupture of membrane (PROM) (group D) at full-term, respectively. Instead of investigating the individual pathogenic role of each lncRNA involved in the molecular mechanism underlying PPROM, we have focused on investigating the metabolic pathways and their functions to explore what is the likely association and how they are possibly involved in the development of PPROM. Six groups, including up-regulation and down-regulation in the comparisons of A vs. B, A vs. C, and A vs. D, of pathways were analyzed. Our results showed that 22 pathways were characterized as up-regulated 7 down-regulated in A vs. C, 18 up-regulated and 15 down-regulated in A vs. D, and 33 up-regulated and 7 down-regulated in A vs. B. Functional analysis showed pathways of infection and inflammatory response, ECM-receptor interactions, apoptosis, actin cytoskeleton, and smooth muscle contraction are the major pathogenic mechanisms involved in the development of PPROM. Characterization of these pathways through identification of lncRNAs opened new avenues for further investigating the epigenomic mechanisms of lncRNAs in PPROM as well as PTB.

## Introduction

Preterm birth (PTB) is a live birth delivered before 37 weeks of gestation (GW), 80% of which are spontaneous (sPTB) [1]. About one-third of PTBs result from the preterm premature rupture of membranes (PPROM) [2]. Although PPROM is usually caused by reproductive genital infections and inflammatory reactions of cytokine and chemokine pathways, and involves the extracellular matrix (ECM) [3]–[5], the pathogenic mechanisms underlying the remaining PTB are not yet well understood. Infections have been associated with and well characterized in PPROM [6], [7]. Approximately 32% of patients with PPROM have amniotic fluid cultures that test positive for microbes at the time of presentation to clinics and 75% of patients have positive cultures at the onset of labor [7]. Proteinases directly secreted by bacteria degrade the collagen in fetal amniotic and chorionic membranes, and in the maternal decidua. Bacteria also produce phospholipase A2 that increases matrix metalloproteinase 1 and 3 (MMP1 and MMP3), leading to ECM degradation [8], [9]. Molecular studies on the development of PPROM have been undertaken [10], with a focus on genes that are involved with inflammation [11]–[14], including TNFα [15]–[17], FAS, which is member 6 gene of the tumor necrosis factor receptor superfamily [9], [18], and Toll-like receptor (TLR) [19], [20]. TNF-α induces apoptosis in PPROM, through binding to the TNF receptor [13]–[15] and activating a proteolytic cascade *via* the FAS-caspase pathway [9]. An apoptotic pathway associated with PPROM involves the binding of p53 to the MMP2 gene promoter [8]. In PPROM, a strong association has been observed between haplotypes of TIMP2 (tissue inhibitors of MMP 2) in maternal DNA and COL4A3 in fetal DNA. A “three-locus” model has been identified in a subset of patients of Hispanic origin in Chile [2]. A similar result was obtained from studying a Norwegian cohort, where haplotypes of COL5A2 in fetal DNA and COL5A1 in maternal DNA were found to associate with spontaneous preterm delivery (PTD) [21]. This suggested there are differing genetic predispositions among different ethnic populations, in agreement with previously reported racial-ethnic disparities [22]. Finding a strong association of single nucleotide polymorphisms with PPROM in a particular ethnic population but not in another one strongly suggests that the heterogeneity and complexity of PPROM is greater than what has yet been perceived. Thus, the molecular mechanisms underlying the pathogenic pathways in PPROM appear to be more complicated. These findings lead us to investigate the complement of pathogenic pathways involved in regulating PPROM.

In recent years there has been an increased focus on noncoding RNAs (ncRNAs). Approximately 98% of total human genomic DNA has been found to be transcribed into ncRNA [23], [24]. Although the roles of many small ncRNAs (such as siRNA and microRNA) are well defined, long chain ncRNA (lncRNA) is much less well characterized. LncRNAs are transcribed RNA molecules greater than 200 nucleotides in length, which are involved in diverse cellular processes such as cell differentiation, imprinting control, immune responses, human diseases and tumorigenesis [25]–[29]. These lncRNAs include not only antisense, intronic transcript and large intergenic ncRNA but also promoter-associated lncRNA and UTR (untranslated region)-associated lncRNA. Knockdown and overexpression studies have shown that an increasing number of lncRNAs play important roles in regulating a diverse spectrum of processes, including splicing [30], transcription [31], localization [32] and organization of subcellular compartments [33]. Underscoring the importance of lncRNAs' regulatory roles is their emergence as key players in the etiology of several disease states [34], [35]. A number of lncRNAs have been demonstrated to alter expression in human cancers and are regulated by specific oncogenic and tumor-suppressor pathways, such as p53, MYC, and NF-κB [36]–[38]. ANRIL is a lncRNA that regulates three separate tumor suppressor genes: p16INK4a, p14ARF and p15INK4b, and is an important negative regulator of the cell cycle [39]. Disruptions to the expression of ANRIL have accordingly been associated with the development of several cancer types, including neuroblastoma [40], acute lymphocytic leukemia [41], melanoma [39] and prostate cancer [42]. Overexpression of the lncRNA transcript encoded by the gene HOTAIR has been associated with hepatocellular carcinoma [43], colorectal cancer [44] and breast cancer [45] by deregulation of HOXD cluster genes. Several lncRNAs have been reported to be important moderators of metabolism and endocrine function, such as PINK1 [PTEN (phosphatase and tensin homologue deleted on chromosome 10)-induced putative kinase 1] [46], H19/IGF2 [47] and thyroid growth receptor α2 (ERBa2) [48]. The regulation of the PINK1 locus is altered in obesity, type II diabetes and inactivity. The Delta 5-desaturase (FADS1) and steroidogenic acute regulatory protein (STAR) genes have also reported lncRNAs [49], [50]. Dreesen et al. demonstrated that the expression of FADS, and its lncRNA, reverses Delta 5-desaturase was found to be reciprocally regulated by dietary fat content in animal models. Moreover, lncRNAs have been reported to involve in other human diseases, such as neurodegenerative and psychiatric diseases [51], cardiovascular disease [52], immune dysfunction and auto-immunity [53]. PPROM is a result of a pathogenic pregnancy. However, there has yet been no study on the possible involvement of lncRNA in PPROM. We hypothesize that lncRNA may play epigenetic/epigenomic function associated with the pathogenic development of PTB including PPROM. In this study, we have initiated an investigation of the differential expression of lncRNAs in placentas of PPROM, compared to controls, and their possible involvement in the pathogenic pathway of PPROM.

## Results

### Identification of lncRNA differentially expressed in PPROM

**Figure 1:**
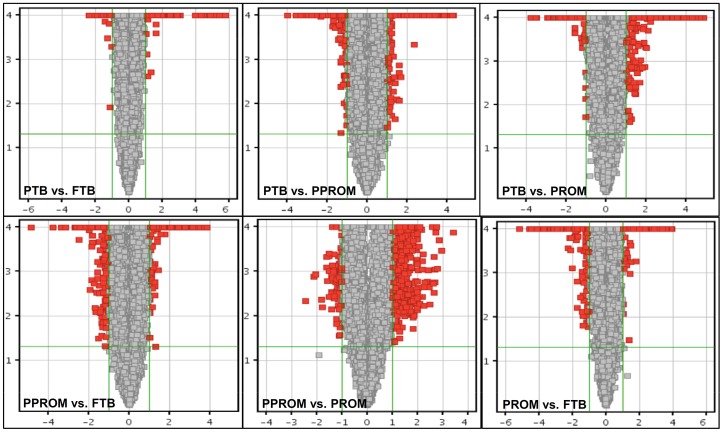
Volcano Plots of six comparisons. X-axis is fold change (log 2) and Y-axis is *p* value (-log 10). Up-regulated (X axis >0) or down-regulated (X axis <0) lncRNAs (red squares) were identified in an about the same number when fold change was set >2 folds [Log 2 (Fold change)] in PTB vs. PPROM. However, there were more down-regulated lncRNAs in PPROM vs. FTB and more up-regulated lncRNAs in PPROM vs. PROM.

A total number of 1968, 1954, and 1050 lncRNAs were identified differentially expressed from placentas of PPROM (group A) compared to FTB (group B), PTB (group C), and PROM (group D), respectively; 449 and 3024 from PTB compared to FTB and PROM, respectively; and 3627 from PROM compared to FTB (Table 1). Differential expression, which was determined by 2-fold change at *p*<0.05, was both up- and down-regulated. Data of differentially expressed lncRNAs, generated by the microarray, has been deposited in Gene Expression Omnibus with an accession number GSE 50879 (http://www.ncbi.nlm.nih.gov/geo/info/linking.html). To visualize differential expression between two different conditions, Volcano Plots (Figure 1) were constructed using fold-change (magnitude of change) values and *p*-values, thus allowing visualization of the relationship between fold-change and statistical significance, which takes both magnitude of change and variability into consideration. The vertical lines correspond to 2.0-fold up and down, respectively, and the horizontal line represents a *p*-value of <0.05. So the red point in the plot represents the differentially expressed lncRNAs with statistical significance. LncRNAs whose *p*-value<0.0001 were assigned to 0.0001, therefore, those points were located in the Y = 4 axis.

**Table 1:** Differential expression of lncRNAs in PPROM.

	A vs. B	A vs. C	A vs. D	C vs. B	C vs. D	D vs. B
**Up-regulated**	**1233**	**974**	**904**	**136**	**1698**	**1245**
**Down-regulated**	**735**	**980**	**146**	**313**	**1326**	**2382**
**Total**	**1968**	**1954**	**1050**	**449**	**3024**	**3627**

A = PPROM, B = FTB, C = PTB, D = PROM.

### Quantitative real-time PCR validation of lncRNAs differentially expressed between PPROM and PTB

**Figure 2:**
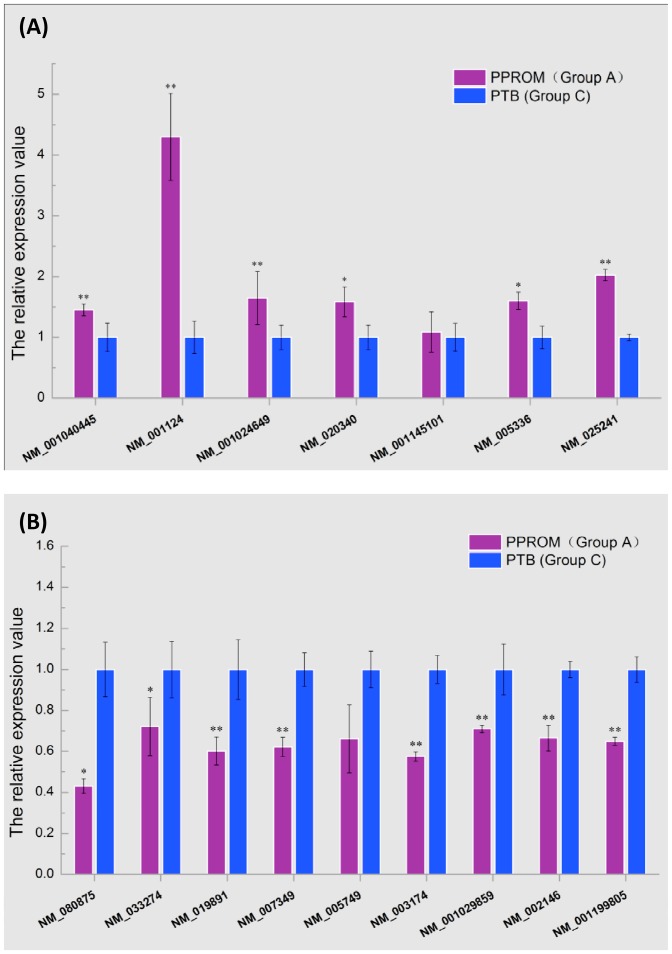
Validation of lncRNAs. RT-qPCR was applied to validate differentially expressed lncRNAs between the PPROM and PTB. Seven up-regulated lncRNAs (A) and nine down-regulated ones (B) were analyzed. Other than the sequence name, each lncRNA was labeled with its associated gene accession in GenBank for a purpose of easy access to its associated gene. Significant levels were indicated by * (*p*<0.05) and ** (*p*<0.01).

Seven up-regulated and nine down-regulated lncRNAs in PPROM (group A) compared with PTB (group C) in microarray assay (Table 2) were randomly chosen for validation, using a technology of real-time quantitative PCR (RT-qPCR). The results revealed that all the up-regulated lncRNAs were increased in PPROM compared to PTB, and six of them rose significantly (Figure 2A). As well, all the down-regulated ones were decreased in PPROM, and eight of them declined significantly (Figure 2B). Therefore, the results of the qPCR analysis were consistent with those of the microarray.

**Table 2:** RT-qPCR validation of differentially expressed lncRNAs.

Differential expression	Name of lncRNA	Associated gene	Primer Sequence (5′-3′)
**Up-regulated**	**AL359337**	**NM_001124**	**F: GCTGTTCGCATATCACCCATT**
			**R: TCTCAGCGAGGTGTAAAGTTGTTC**
	**uc002vyh.2**	**NM_001040445**	**F: TCCCTCAGAGCAGGTGAAAAC**
			**R: ACAGGTGGAAGTGTTGGTCGT**
	**G65718**	**NM_020340**	**F: GCGCAGTTAGCAGCAACG**
			**R: GAACCAGAAGGAGCGGGAC**
	**AK000995**	**NM_001145101**	**F: CAGCAGCACTTGTGATTCGG**
			**R: GGAACCTATGTTGGCTATTGACTG**
	**CR604135**	**NM_001024649**	**F: ACATATACTGTCAACGGAGGGTG**
			**R: TGTGTGGGATCATTGCATTTC**
	**G42939**	**NM_025241**	**F: CAGGTCTATGTGCAGGAAGGC**
			**R: GCTGCTCTGTGGATGTGGG**
	**AF116718**	**NM_005336**	**F: CTCCACGGGAAACCCATAAC**
			**R: GTGTTCAGTTGCCTTCTTTCTACC**
**Down-regulated**	**BC062331**	**NM_001029859**	**F: TCCCTCTTGGTGGGCATCT**
			**R: CAACCTCACTGGCGACCCT**
	**G36711**	**NM_033274**	**F: GTTTTCCGTGAACAATCTCCC**
			**R: GAACGCCAACTGGAAAGGTC**
	**AK056945**	**NM_080875**	**F: TGCCAGAGCGGAGAACGA**
			**R: CGGGTCCCACAGTTTCCAG**
	**nc-HOXB2-164**	**NM_002146**	**F: AGATGGAGGCAGCAGAATGG**
			**R: TTTAGAGGGCTCAGGGATGGT**
	**uc001qya.1**	**NM_001199805**	**F: GTAAGTTTACCTAGTGCTGCTCCC**
			**R: TGACCGCTAATCAAGTCTGTCC**
	**NR_024476**	**NM_007349**	**F: CCAGTGGCAATGTCCAAGAAG**
			**R: TCAGCAAACTGGTCCATACGC**
	**BC015064**	**NM_005749**	**F: ATGAGGCTGGGTCACTTATGG**
			**R: TGATGTTCGTGGCAATCTGC**
	**AK092850**	**NM_003174**	**F: CAGCTTCTGGGTGGAGCATT**
			**R:TCGTTCGCCGTCAGTCATT**
	**GAPDH**	**M88109**	**F: GGGAAACTGTGGCGTGAT**
			**R: GAGTGGGTGTCGCTGTTGA**

### Metabolic pathways involved in PPROM

**Figure 3:**
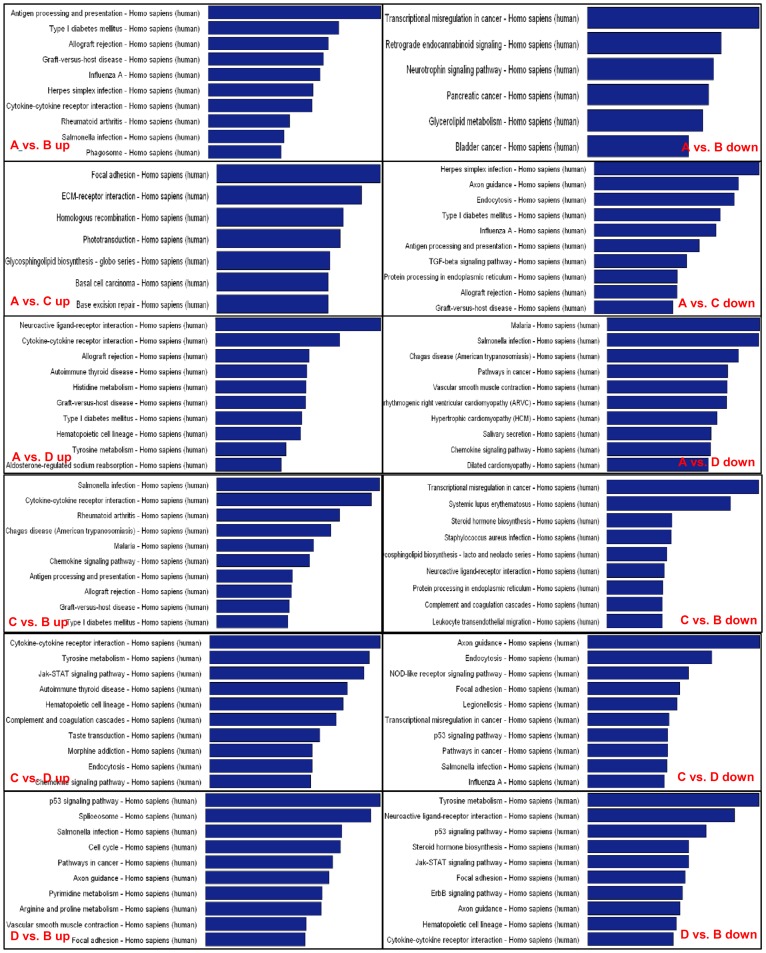
Metabolic pathways characterized from the lncRNAs differentially expressed in PPROM. Six groups of pathways (each group has up- and down-regulated) were characterized with KEGG functional analysis. Three p values, the EASE-score, Fisher-Pvalue and Hypergeometric-Pvalue were integrated for the analysis. The bar plot shows the top Enrichment Score [-log10(Pvalue)] value of the significant enrichment pathway. If there were more than 10 pathways whose Enrichment Score is >0.05, only the top 10 pathways are shown here. The higher Enrichment Score indicates the more lncRNA molecules are involved in this pathway.

A functional analysis of mapping genes to KEGG Pathways was performed with a *p*-value = 0.05 as cut-off. The *p*-value (EASE-score, Fisher-Pvalue or Hypergeometric-Pvalue) denotes the significance of the Pathway correlated to the conditions. The lower the *p*-value, the more significant is the Pathway. Six groups of pathways were analyzed (Table 3), including up-regulation and down-regulation in the comparison of PPROM vs. FTB (A vs. B), PPROM vs. PTB (A vs. C), PPROM vs. PROM (A vs. D), PTB vs. FTB (C vs. B), PTB vs. PROM (C vs. D), and PROM vs. FTB (D vs. B). Our results showed that 34 pathways were characterized as up-regulated 6 down-regulated in A vs. B, 7 up-regulated and 22 down-regulated in A vs. C, 18 up-regulated and 15 down-regulated in A vs. D; 16 up-regulated and 9 down-regulated in C vs. B, 12 up-regulated and 26 down-regulated in C vs. D; and 28 up-regulated and 15 down-regulated in D vs. B. Pathways with the Top 10 enrichment scores (preset *p*<0.05 as cutoff) were shown in Figure 3. When PPROM was compared to PTB (A vs. C), rupture of membrane was the focus; while if compared to PROM (A vs. D), the preterm was the focus. If PPROM compared to FTB, both rupture of membrane and preterm labor would be analyzed.

**Table 3:** PPROM pathways identified from differentially expressed lncRNAs.

	Number of pathway: Up-regulated	Number of pathway: Down-regulated
**A vs. B**	**22: Infection-inflammatory**	**2: Apoptosis**
	**5: Signaling**	**2: Signaling**
	**3. Cell metabolism**	**1: Misregulation of transcription**
	**1: RNA transport**	**1: Glycerolipid metabolism**
	**1: Protein processing**	**1: ECM-receptor interaction**
	**1: Regulation of actin cytoskeleton**	
**A vs. C**	**2: DNA synthesis**	**11: Infection-inflammatory**
	**2: Signal transduction**	**4: Signaling**
	**1: Glycosphingolipid biosynthesis**	**2: Metabolism**
	**1: Focal adhesion**	**2: Neurological related**
	**1: ECM-receptor interaction**	**1: RNA transport**
		**1: Protein processing**
		**1: Regulation of actin cytoskeleton**
**A vs. D**	**11: Infection-inflammatory**	**9: PLC and IL8 involved Immuno-inflammatory**
	**4: Metabolism**	**5: Smooth muscle contraction**
	**1: Cell adhesion molecules**	**1: ECM-receptor interaction**
	**1: Aldosterone-regulated sodium reabsorption**	
	**1: Hematopoietic cell linage**	
		
**C vs. B**	**11: Infection-inflammatory**	**3: Infection-inflammatory**
	**3: Metabolism**	**3: Metabolism**
	**1: Cell adhesion molecules**	**1: Neuroactive ligand-receptor interaction**
	**1: Complement and coagulation cascades**	**1: Complement and coagulation cascades**
		**1: Transcriptional misregulation**
		
**C vs. D**	**5: Signaling**	**13: Infection-inflammatory**
	**4: Infection-inflammatory**	**6: Signaling**
	**1: Neuroactive ligand-receptor interaction**	**4: Metabolism**
	**1: Complement and coagulation cascades**	**1: Smooth muscle contraction**
	**1: Hematopoietic cell lineage**	**1: Transcriptional misregulation**
		**1: Cell adhesion molecules**
**D vs. B**	**10: Metabolism**	**5: Signaling**
	**6: Smooth muscle contraction**	**4: Metabolism**
	**4: Signaling**	**2: Infection-inflammatory**
	**4: Infection-inflammatory**	**2: Cell adhesion molecules**
	**3: Cell adhesion molecules**	**1: Smooth muscle contraction**
	**1: ECM-receptor interaction**	**1: Neuroactive ligand-receptor interaction**

A = PPROM, B = FTB, C = PTB, D = PROM.

Infection-inflammatory pathways were the most common ones identified in the up-regulated condition, although they were in the down-regulated condition in A vs. D. The ECM (extracellular matrix) interacted with the receptor present in all the down-regulated groups. This suggested that infection-inflammatory and degradation of ECM were the two major pathogenic mechanisms associated with PPROM, in agreement with previous studies [1], [3], [5], [7]–[9]. Up-regulated actin cytoskeleton pathway suggested that lncRNA interferes with the normal structure and function of the cytoskeleton. The smooth muscle contraction pathway in the down-regulation of A vs. D strongly indicated that reduced expression of lncRNAs in the smooth muscle contraction pathway is the major driving factor underlying the rupture of membrane. This could increase the placental smooth muscle contraction inducing the premature labor, in addition to damage of intake membrane by ECM-receptor interaction.

### GO molecular function annotations

**Figure 4:**
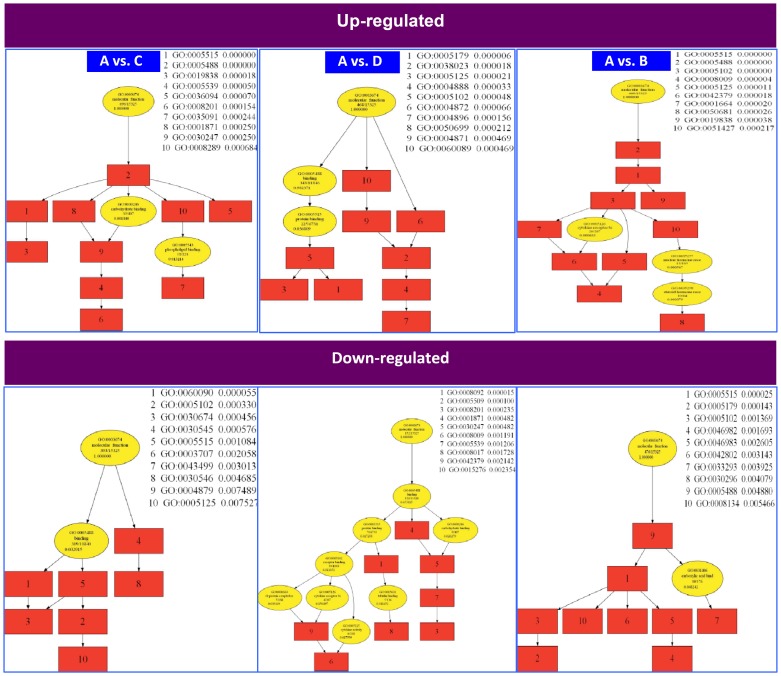
GO molecular functional annotations. Functional relationship presented with GO “trees” for the top 10 (*p*<0.005) GO annotations (Table 3) is shown for both up- and down-regulated A vs. B, A vs. C, and A vs. D. The red color labels indicate the top 10 GOs. The yellow colors show GOs with lower *p* value, which were not presented in Table 3 but could be identified from the differentially expressed lncRNAs in PPROM, FT, PRTB, and PROM.

The Gene Ontology (GO) website (http://www.geneontology.org) provides a controlled vocabulary to describe gene and gene product attributes in any organism. The ontology covers three domains: Biological Process, Cellular Component and Molecular Function. Fisher's exact test was used to determine if there is more overlap between the list of PPROM vs. controls and that of GO annotation than expected by chance. The *p*-value denotes the significance of GO terms enrichment in the A vs. C, A vs. D, and A vs. B genes. The lower the *p*-value (*p*-value<0.05 was applied), the more significant is the GO Term. Top 10 gene ontology (GO) molecular function annotations for lncRNAs (Table 4), based on their *p* values, showed their relative relationship with each other (Figure 4). Each color labeled box or circle represents a GO functional pathway. Red color labels indicate that these GO pathways are associated with PPROM and yellow labels were not detected from PPROM in this study. The order of the number represents the p value for how significant it is associated with PPROM.

**Table 4:** GO molecular function annotations in PPROM.

	Regulation	GO number	P value
**A vs. B**	**Up-**	**1.GO:0005179**	**0.000006**
		**2.GO:0038023**	**0.000018**
		**3.GO:0005125**	**0.000021**
		**4.GO:0004888**	**0.000033**
		**5.GO:0005102**	**0.000048**
		**6.GO:0004872**	**0.000066**
		**7.GO:0004896**	**0.000156**
		**8.GO:0050699**	**0.000212**
		**9.GO:0004871**	**0.000469**
		**10.GO:0060089**	**0.000469**
	**Down-**	**1.GO:0008092**	**0.000015**
		**2.GO:0005509**	**0.000100**
		**3.GO:0008201**	**0.000235**
		**4.GO:0001871**	**0.000482**
		**5.GO:0030247**	**0.001191**
		**6.GO:0008009**	**0.001206**
		**7.GO:0005539**	**0.001728**
		**8.GO:0008017**	**0.000212**
		**9.GO:0042379**	**0.002142**
		**10.GO:0015276**	**0.002345**
**A vs. C**	**Up-**	**1.GO:0005515**	**0.000000**
		**2.GO:0005488**	**0.000000**
		**3.GO:0019838**	**0.000018**
		**4.GO:0005539**	**0.000050**
		**5.GO:0036094**	**0.000070**
		**6.GO:0008201**	**0.000154**
		**7.GO:0035091**	**0.000244**
		**8.GO:0001871**	**0.000250**
		**9.GO:0030247**	**0.000250**
		**10.GO:0008289**	**0.000684**
	**Down-**	**1.GO:0060090**	**0.000055**
		**2.GO:0005102**	**0.000330**
		**3.GO:0030674**	**0.000456**
		**4.GO:0030545**	**0.000576**
		**5.GO:0005515**	**0.001084**
		**6.GO:0003707**	**0.002058**
		**7.GO:0043499**	**0.003013**
		**8.GO:0030546**	**0.004685**
		**9.GO:0004879**	**0.007489**
		**10.GO:0005125**	**0.007527**
**A vs. D**	**Up-**	**1.GO:0005515**	**0.000000**
		**2.GO:0005488**	**0.000000**
		**3.GO:0005102**	**0.000000**
		**4.GO:0008009**	**0.000004**
		**5.GO:0005125**	**0.000011**
		**6.GO:0042379**	**0.000018**
		**7.GO:0001664**	**0.000020**
		**8.GO:0050681**	**0.000026**
		**9.GO:0019838**	**0.000038**
		**10.GO:0051427**	**0.000217**
	**Down-**	**1.GO:0005515**	**0.000025**
		**2.GO:0005179**	**0.000143**
		**3.GO:0005102**	**0.001369**
		**4.GO:0046982**	**0.001693**
		**5.GO:0046983**	**0.002605**
		**6.GO:0042802**	**0.003143**
		**7.GO:0033293**	**0.003925**
		**8.GO:0030296**	**0.004079**
		**9.GO:0005488**	**0.004880**
		**10.GO:0008134**	**0.005466**

A = PPROM, B = FTB, C = PTB, D = PROM.

### LncRNAs shared by two or more conditions

To analyze all lncRNA molecules among the six groups of comparisons, the following lncRNAs involved in PPROM were identified.

- Cytokine-cytokine receptor interaction pathway and rheumatoid arthritis pathway were shown in both up- and down-regulated when A vs. C. However, the lncRNA molecules identified from PPROM in these two pathways were not the same at up- or down-regulation.
- Rad51 and Shc were shared in down-regulation of A vs. C and A vs B.
- BMP, ECM, TCF/LEF were shared by A vs. C (down-regulation) and A vs. D (down-regulation).
- IFNγ, MHCI, and/or MHCII were shared by the groups of all up-regulation of A vs. B, vs. C, and vs. D.
- CLDN, EGFR, IFNγ, MHCI, MHCII, MHCI/II were shared by up-regulation of A vs. C and A vs. D.
- CD3, CXCL9, CXCL10, FLT1, GZM, GZMB, HLA-DR, IFNG, IFNγ, IL1RAP, IL2RB, MHCI, MHCII, MHCI/II were shared by up-regulation of A vs. B and A vs. D.
- AP1, CASP1, CCL2, CHMP3, CHMP4, cofilin, CTSB/L/S, Cyclin, Cyclin D, DUB, EEA1, eIF1, eIF3, eIF4g, eIF4γ, EphA, E3ligase, FIP, FNR, GPCR, HLA-C, HLA-G1, hnRNP K, Hsc70, HSP70, HSP72, HSP90, IA-2, IFNAR, IFNAR2, IFNγ, IFNs, IFNsR, IL-2R, IP10, IRF9, ITGB1, JAK1/Tyk2, JAK1/2, JAK1/3, LARG, LTBP1, MCP1, MHCI, MHCII, MHCI/II, NFAT, Np14, OSTs, PERK, PKR, p107, p120, Rab5, Rab22, Ras, GAP, ROCK, ROCK1, RTK, Sec62/63, Sema6, Smurf1/2, Smurf2, SRPK1, STAM, SUMO, TAP1/2, THOC2, TNFα, TRAIL, TRAM, Upf2, Upf3, Wnt have been determined as the up-regulation of A vs. B and A vs. C.

### Differential expression of lncRNAs embedded in coding sequence

Thirteen differentially expressed lncRNAs have been identified by the criteria of fold ≥2 changed and *p*<0.05 (Table 5). These lncRNAs were embedded in coding regions. Seven were presented in the A vs. C, six in A vs. D, and seven in A vs. B. There was an up-regulated antisense lncRNA, embedded in the AKAP8 locus, which was an unique one present only in the group of A vs. C; similarly for the up-regulated sense lncRNA of QSOX1 and antisense lncRNA of SYNPO2L, ADAM12, and ADH6 in the group of A vs. D. Two down-regulated lncRNAs, TP73 and HOMER3, were identified in the group of A vs. B only. The remaining lncRNAs were presented in two or three groups (Table 5).

**Table 5:** Differentially expressed lncRNA in coding sequences.

	A vs. C	A vs. D	A vs. B	Accession	Gene	Strand	Protein
**Up-regulated**	**5 5.73216**	**5.610379649**	**2.984750315**	**NM_002127**	**HLA-G**	**+**	**Major histocompatibility complex, class I, G**
	**4.85154**	**1.062784137**	**1.958096274**	**NM_001003681**	**HMGXB4**	**+**	**HMG box domain containing 4**
	**4.631371**	**1.017124111**	**2.073246461**	**NM_003156**	**STIM1**	**+**	**Stromal interaction molecule 1**
	**1.245048**	**2.495066469**	**1.140838006**	**NM_001004128**	**QSOX1**	**+**	**Quiescin Q6 sulfhydryl oxidase 1**
	**3.57514**	**0.937050789**	**2.73985824**	**NM_012215**	**MGEA5**	**-**	**Meningioma expressed antigen 5 (hyaluronidase)**
	**3.53793**	**0.965556342**	**1.820589859**	**NM_005858**	**AKAP8**	**-**	**A kinase (PRKA) anchor protein 8**
	**1.15697**	**3.411582277**	**0.877613084**	**NM_001114133**	**SYNPO2L**	**-**	**Synaptopodin 2-like**
	**1.10173**	**2.908425097**	**1.198747287**	**NM_003474**	**ADAM12**	**-**	**ADAM metallopeptidase domain 12**
	**0.793904**	**2.295361844**	**0.664734368**	**NM_000672**	**ADH6**	**-**	**Alcohol dehydrogenase 6 (class V)**
**Down-regulated**	**0.4410479**	**0.498847611**	**0.295686434**	**NM_004100**	**EYA4**	**+**	**Eyes absent homolog 4 (Drosophila)**
	**0.617984**	**1.086721628**	**0.481554083**	**NM_001126240**	**TP73**	**+**	**Tumor protein p73**
	**0.4626384**	**1.349994706**	**0.380171464**	**NM_002632**	**PGF**	**-**	**Placental growth factor**
	**0.616802**	**1.13796367**	**0.482993323**	**NM_001145724**	**HOMER3**	**-**	**Homer homolog 3 (Drosophila)**

A = PPROM, B = FTB, C = PTB, D = PROM.

## Discussion

Previous studies of PPROM have suggested it is associated with genital infections and inflammatory reactions of cytokine and chemokine pathways, and involves the extracellular matrix (ECM). However, the molecular pathogenic mechanisms underlying the pathogenic pathways in PPROM appear to be more complicated, and the involvement of the complement of pathogenic pathways in regulating PPROM has been unclear. Recently, increasing evidence has confirmed lncRNAs are one of the most important factors controlling gene expression [54], and they have important roles in imprinting control, cell differentiation, immune responses, human diseases, tumorigenesis and other biological processes [25], [26], [55]–[57]. In this study, we identified 1,968 lncRNAs that were differentially expressed in PPROM when compared to FTB, 1954 in PPROM when compared to PTB, and 1,050 in PPROM when compared to PROM. To our knowledge, there is no report up to now describing lncRNAs expression in either normal or pathological placentas, and there has been no study on the association of differentially expressed lncRNA with the molecular mechanisms underlying the pathogenic pathways in preterm birth including PPROM.

lncRNAs have been recognized to have comprehensive functions in biological processes through various mechanisms [58], and to play important roles in both normal development and disease [59]. The lncRNAs that have been identified may be involved in the development and progression of PPROM and provide novel approaches to better understand the molecular basis of PPROM. Almost every step in the life cycle of genes—from transcription to mRNA splicing, RNA decay, and translation—can be influenced by lncRNAs [34]. Through distinct mechanisms by which lncRNAs regulate gene expression, they may regulate different metabolic pathways and participate in a wide repertoire of biological processes [58]. In this study, we identified lncRNAs that are localized in untranslated regions (UTRs) and embedded in protein-coding regions in human placentas from patients with PPROM (group A) and compared them to FTB (group B), PTB without premature rupture of membranes (group C), and PROM (group D). Comprehensive in-depth analysis of the expression profiles of lncRNAs in these group comparisons (A vs. B, A vs. C, A vs. D, C vs. B, C vs. D, and D vs. B) was carried out in order to understand the role of lncRNAs in PPROM, which may help in uncover both preterm delivery and rupture of membrane.

To validate the reliability of microchip technology, 16 lncRNAs, including 7 up-regulated and 9 down-regulated, that represent the differential expression of lncRNAs in PPROM were randomly selected and subjected to RT-qPCR validation (Table 2). Our results demonstrated that the differential expression of 86% (6 out of 7) up-regulated and 89% (8 out of 9) down-regulated lncRNAs were statistically significant (Figure 2). Among the significantly expressed 14 lncRNAs, 10 (71%) were significant at p<0.001 and 4 (29%) were at p<0.05. 12.5% (2 out of 16) lncRNAs, one from each up-regulated and down-regulated group, were not significant although the RT-qPCR showed their expression was strikingly altered. This provided an overall concordance of 87.5% of the lncRNAs identified from this study with the microchip technology to be reliable. We believe that an increase of the sample size of validation would increase the analysis power and that a high degree of reliability may be achieved.

Instead of investigating the individual pathogenic function of each lncRNA involved in the pathogenic mechanism underlying PPROM, we have focused on investigating the biological pathways and their functions in order to explore what is the likely association and how they are possibly involved in the development of PPROM. Our results showed (at *p*≤0.05) that there were 29 biological pathways, including 7 up-regulated and 22 down-regulated, in comparisons of PPROM vs. PTB (A vs. C); 33 pathways, including 18 up-regulated and 15 down-regulated, identified in A vs. D (PROM), and 40 pathways, including 33 up-regulated and 7 down-regulated, in A vs. B (FTB). Because both PPROM and PTB are delivered at ≤35 gestational weeks, the only variation between these two groups was the premature rupture of membrane (PROM). From this comparison, our attention was focused on the lncRNA(s) that are involved in membrane rupture, which could be a result of premature labor [60], different from the D vs. B where the rupture is the primary factor. However, when PPROM was compared to PROM, the focus was preterm labor since both PPROM and PROM share the common feature of premature membrane rupture. Normal birth at 39–40 gestational weeks without rupture of membrane provided two variation factors for PPROM, which are both preterm and membrane rupture. Therefore, A vs. B may show a combination of pathways presented in both A vs. C and A vs. D. In addition, we have shown 38 pathways in C vs. B, 38 pathways in C vs. D, and 43 pathways in D vs. B, which provided more detailed information to complement the PPROM pathways (Table 3).

Among the PPROM pathways, immunoreactions related to infection and inflammation, including chemokine and cytokine pathways, were the most common pathways within the top-10 list (Figure 3), which were up-regulated in all A vs. C, A vs. B, and A vs. D comparisons and in down-regulated A vs. D. This supported and explained the consensus of most researchers that PPROM is usually caused by genital infections, inflammatory reactions of cytokine and chemokine pathways [61], [62]. In the immune processes caused by pathogenic infections, leukocytes secrete different cytokines, among which pro-inflammatory cytokines (including IL-1β, IL-6 and TNF-α) activate host inflammatory response and were closely associated with PPROM [62], [63]. TNF-α can bind the receptor TNFR and induce activation of apoptosis, MMPs (matrix metalloproteinases) and caspase, and degradation of extracellular matrix (ECM) in fetal membranes, which play a major role in promoting PPROM [62]. Infection and inflammatory pathway is also seen in the list of top-10 pathways of non-PPROM groups, in both up- and down-regulation, although it may not be the most common pathway in the group of C vs. D and D vs. B.

Pathway of ECM-receptor interaction is presented in all PPROM down-regulated comparisons in this study, although there was only one pathway in each comparison. PPROM was closely associated with the remodeling of ECM, because weakening of the amniochorion ECM is one of the key events predisposing to membrane rupture [62], [64]. The down-regulated focal adhesion and ECM-receptor interaction may further provide evidence supporting the previous clue [62], [64] that the remodeling of ECM is a key component in the pathogenesis of PPROM. In this study, ECM-receptor interactions were involved in down-regulation of lncRNAs of collagens, laminin, OPN, VLAα10, α/βDG, and α6β1, which suggested that these lncRNAs may be critical for the decreased synthesis of mRNA and resulted in weakness of the extra cellular matrix. Up-regulation of lncRNA involved in ITGA and ITGB suggested a regulatory function that increased lncRNA could reduce the synthesis of ITGA and ITGB. The up-regulated pathway of ECM-receptor interaction in A vs. C and D vs. B but down-regulated in A vs. B and A vs. D may suggest different mechanisms of regulation that lncRNAs involved, which deserved further investigations.

**Figure 5:**
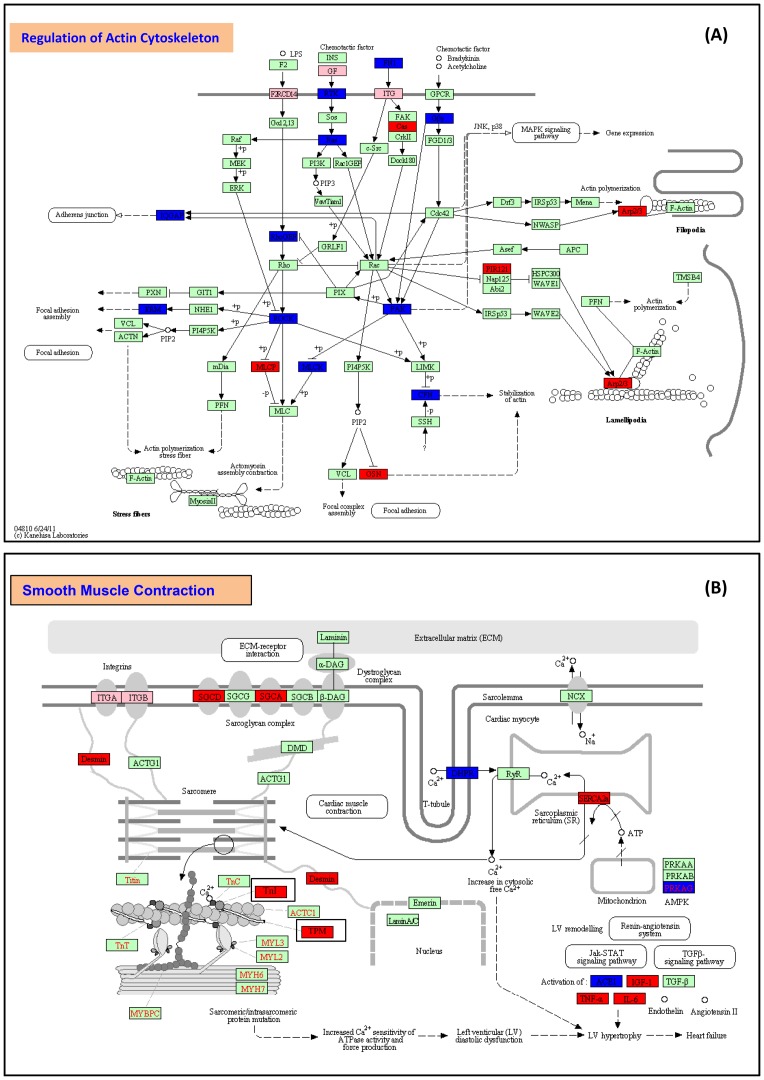
Pathway diagrams. (A) A diagram of actin cytoskeleton pathway showed all lncRNA molecules identified from A vs. C. Red labels are up-regulated and blue labels are down-regulated lncRNAs at *p*<0.01. Pink labels are the lncRNAs up-regulated at *p*<0.05. Green colors are detected by Arraystar microchip without significance at *p*>0.05. (B) A diagram of smooth muscle contraction pathway showed all lncRNA molecules identified from A vs. D. Red labels are up-regulated and blue labels are down-regulated lncRNAs at *p*<0.01. Pink labels are the lncRNAs up-regulated at *p*<0.05. Green colors are detected by Arraystar microchip without significance at *p*>0.05.

Although the second most common pathway in PPROM is the signaling since there were 13 pathways involved in both up- and down-regulation of A vs. C and A vs. B, the pathways of regulation of actin cytoskeleton in A vs. C and that of smooth muscle contraction in A vs. D are more relevant to the premature labor. LncRNAs of ERM, IQGAP, ROCK, RhoGEF, F2RCD14, GF, RTK, Ras, FN1, ITG, MLCK, PAK, CFN, and Gδγ identified from our discovery study of microarray were differentially expressed in the pathway regulating actin cytoskeleton, through activation of actin stress fiber, actin polymerization, and actomyosin assembly contraction in addition to adherens junction, focal adhesion, and MARK signaling (Figure 5A). In A vs. D, the regulation of lncRNA on smooth muscle contraction could be through calcium signaling by PLC, s-GC, CRLR, MHC, DHPR, TnI, TPM, ACTAC1RyR2, and TCF/LEF; or by ECM-receptor interactions through SGCD, Desmin, and Lamin A/C, as shown in an example of the pathway in Figure 5B.

Thirteen differentially expressed lncRNAs embedded in coding sequences, among which, six are sense strand and seven are antisense strand, have been identified (Table 5). The lncRNA embedded in the coding locus of AKAP8 was the one identified from A vs. C only; QSOX1, SYNPO2L, ADAM12, and ADH6 were unique from A vs. D; and TP73 and HOMER3 were unique for A vs. B [65]–[68]. These unique differentially expressed lncRNA molecules may lead us to further explore their possible pathogenic function in the development PPROM. Two antisense lncRNAs were particularly interesting in this study, the ADAM12 (ADAM metallopeptidase domain 12) [69], [70] and PGF (placental growth factor) [71]. ADAM12 (a disintegrin and metalloprotease) is a member of a family of multidomain proteins with structural homology to snake venom metalloproteases. This family possesses extracellular metalloprotease and cell-binding functions, as well as intracellular signaling capacities. It is an active metalloprotease, and has been implicated in insulin-like growth factor (IGF) receptor signaling, through cleavage of IGF-binding proteins, and in epidermal growth factor receptor (EGFR) pathways, *via* ectodomain shedding of membrane-tethered EGFR ligands. These proteolytic events may regulate diverse cellular responses, such as altered cell differentiation, proliferation, migration, and invasion. ADAM12 may also regulate cell-cell and cell-extracellular matrix contacts through interactions with cell surface receptors—integrins and syndecans—potentially influencing the actin cytoskeleton. Interacting with several cytoplasmic signaling and adaptor molecules through its intracellular domain, ADAM12 thereby directly transmits signals to or from the cell interior [69], [70]. These ADAM12-mediated cellular effects appear to be critical events in both biological and pathological processes in PPROM, as well as in PROM and PTB. The PGF was identified from A vs. B and A vs. C. It is a placental vascular endothelial growth factor-related protein, activating angiogenesis and endothelial cell growth, stimulating their proliferation and migration. It binds to the receptor FLT1/VEGFR-1 [71]. It is likely that reduction of lncRNA would reduce the mRNA of PGF in the placenta and participate in the PPROM. Characterization of these coding region embedded lncRNAs may have laid a base for further investigating the molecular mechanisms of how lncRNAs may be involved in regulating the pathways associated with PPROM, PTB and/or PROM. Further validation and functional study with focusing on the lncRNAs regulation on the genes discussed here are necessary to illustrate their mechanism(s) of regulation.

In summary, identification of thousands of differentially expressed lncRNAs from the human placentas of PPROM, FTB, PTB, and PROM provides evidence that lncRNAs may be participating in the physiological and pathogenic processes of human pregnancies relevant to reproductive conditions and disorders. Characterization of metabolic pathways further supports previous findings that infection and inflammatory response, ECM-receptor interactions, apoptosis and smooth muscle contraction are the major pathogenic mechanisms involved in the development of PPROM, along with PROM and PTB. Although the detailed function and pathogenesis how individual lncRNAs play their role(s) in the PPROM and PTB is still unknown, our findings have opened a new avenue for exploration.

## Materials and Methods

### Ethics statement

The Hospital Ethics Committee reviewed and approved the research project. Informed consent was obtained from all participants. All materials and data were previously encoded and kept anonymous to the authorship of this study.

### Placentas

A total 40 placentas from age-matched (25–30 years old) were divided into four groups (10 placentas per group) of deliveries. These are: group A, PPROM ≤35 weeks of gestation; group B, FTB at 39–40 weeks of gestation without membrane rupture; group C, PTB at ≤35 weeks of gestation without membrane rupture; and group D, PROM (premature membrane rupture) at 39–40 weeks of gestation. Placentas were flushed twice with 200ml cold distilled water that was absorbed with clean paper towels, sliced with a sterile scalpel into 1×1 cm^2^ cubes, juxtaposed to the fetal membrane, quickly frozen in liquid nitrogen for 30 minutes, and stored at −80°C until use. The entire procedure should be completed within 30 minutes after the placenta is delivered.

### Microarray

The Arraystar Human LncRNA Array v2.0 was designed for researchers who were interested in profiling lncRNAs that are localized at UTRs and embedded in protein-coding regions in the human genome. 33,045 lncRNAs were collected from the authoritative data sources including RefSeq, UCSC Knowngenes, Ensembl and many related articles from the literature (www.arraystar.com).

### RNA labeling and array hybridization

Sample labeling and array hybridization were performed according to the Agilent One-Color Microarray-Based Gene Expression Analysis protocol (Agilent Technology, Santa Clara, CA) with minor modifications. Briefly, mRNA was purified from total RNA after removal of rRNA with mRNA-ONLY™ Eukaryotic mRNA Isolation Kit (Epicentre, Omaha, NE). Then, each sample was amplified and transcribed into fluorescent cRNA along the entire length of the transcripts without 3′ bias utilizing a random priming method. The labeled cRNAs were purified by RNeasy Mini Kit (Qiagen, Valencia, CA). The concentration and specific activity of the labeled cRNAs (pmol Cy3/µg cRNA) were measured by NanoDrop ND-1000. 1 µg of each labeled cRNA was fragmented by adding 5 µl 10× Blocking Agent and 1 µl of 25× Fragmentation Buffer, then heated the mixture at 60°C for 30 min, finally 25 µl 2×GE Hybridization buffer was added to dilute the labeled cRNA. 50 µl of hybridization solution was dispensed into the gasket slide and assembled to the LncRNA expression microarray slide. The slides were incubated for 17 hours at 65°C in an Agilent Hybridization Oven. The hybridized arrays were washed, fixed and scanned with using the Agilent DNA Microarray Scanner (Agilent Technology, Santa Clara, CA).

### Data analysis

Agilent Feature Extraction software (version 11.0.1.1) was used to analyze acquired array images. Quantile normalization and subsequent data processing were performed with using the GeneSpring GX v12.1 software package (Agilent Technologies, Santa Clara, CA). After normalization of the raw data, lncRNAs and mRNAs that have flags (“All Targets Value”) were chosen for further data analysis. Differentially expressed lncRNAs and mRNAs with statistical significance between the two groups were identified through Volcano Plot filtering. Hierarchical Clustering was performed using the Agilent GeneSpring GX software (Version 12.1). Both “GO analysis” and “Pathway analysis” were performed in the standard enrichment computation method.

### Quantitative real-time PCR analysis

Total RNA was extracted from placentas, and cDNA was synthesized. The expression level of lncRNAs were determined by qPCR, and the primer sequences were listed in Table 2. qPCR reactions were performed by the ABI7900 system (Applied Biosystems) and SYBR green dye PCR master mix (SuperArray). Glyceraldehyde 3-phosphate dehydrogenase (GAPDH) was used as an internal control, and lncRNAs' values were normalized to GAPDH. For each lncRNA, the result was finally reported as relative expression by setting the expression value in PTB (group C) at 1 and the expression value in PPROM (group C) was calculated relative to this control. All data were given in terms of relative expression of mean ± S.E. (N = 5). The data were subjected to one-way analysis of variance (one-way ANOVA) followed by an unpaired, two-tailed t-test. Differences were considered significant at *P*<0.05 (labeled as “ * ” in Figure 2) and extremely significant at *P*<0.01 (labeled as “ ** ” in Figure 2).
